# Endogenous glutamine production in critically ill patients: the effect of exogenous glutamine supplementation

**DOI:** 10.1186/cc13829

**Published:** 2014-04-14

**Authors:** Maiko Mori, Olav Rooyackers, Marie Smedberg, Inga Tjäder, Åke Norberg, Jan Wernerman

**Affiliations:** 1 Department of Anaesthesia and Intensive Care Medicine, Karolinska University Hospital Huddinge and Karolinska Institutet, 14186 Stockholm, Sweden

## Abstract

**Introduction:**

Glutamine rate of appearance (R_a_) may be used as an estimate of endogenous glutamine production. Recently a technique employing a bolus injection of isotopically labeled glutamine was introduced, with the potential to allow for multiple assessments of the glutamine R_a_ over time in critically ill patients, who may not be as metabolically stable as healthy individuals. Here the technique was used to evaluate the endogenous glutamine production in critically ill patients in the fed state with and without exogenous glutamine supplementation intravenously.

**Methods:**

Mechanically ventilated patients (n = 11) in the intensive care unit (ICU) were studied on two consecutive days during continuous parenteral feeding. To allow the patients to be used as their own controls, they were randomized for the reference measurement during basal feeding without supplementation, before or after the supplementation period. Glutamine R_a_ was determined by a bolus injection of ^13^C-glutamine followed by a period of frequent sampling to establish the decay-curve for the glutamine tracer. Exogenous glutamine supplementation was given by intravenous infusion of a glutamine containing dipeptide, L-alanyl-L-glutamine, 0.28 g/kg during 20 hours.

**Results:**

A 14% increase of endogenous glutamine R_a_ was seen at the end of the intravenous supplementation period as compared to the basal measurements (*P* = 0.009).

**Conclusions:**

The bolus injection technique to measure glutamine R_a_ to estimate the endogenous production of glutamine in critically ill patients was demonstrated to be useful for repetitive measurements. The hypothesized attenuation of endogenous glutamine production during L-alanyl-L-glutamine infusion given as a part of full nutrition was not seen.

## Introduction

Glutamine depletion as indicated by a low plasma glutamine concentration at ICU admittance is an independent predictor of an unfavorable outcome [1,2]. Exogenous glutamine supplementation is the standard of care when parenteral nutrition is required in critically ill patients [3,4]. Advantages in terms of mortality and morbidity have been demonstrated when the exogenously supplemented glutamine is administered intravenously [3]. In a recent study, however, harm was reported when pharmacological doses of glutamine were administered to critically ill patients combined with hypocaloric feeding [5]. The existence of conflicting clinical data is a strong argument to systematically elucidate how exogenous glutamine supplementation is handled, in particular the relation between plasma concentrations and the endogenous glutamine production.

Glutamine rate of appearance (R_a_) may be used as an estimate of endogenous glutamine production. There are several ways to measure and calculate glutamine R_a_; by a constant infusion of isotopically labeled glutamine [6-9], by the use of increment curves or decay curves in conjunction with the start or end of a constant intravenous infusion of exogenous glutamine [10,11], and recently by a bolus injection of isotopically labeled glutamine [12]. These techniques show comparable results and may have inherent advantages or disadvantages when utilized in various clinical situations. The bolus injection technique allows for repeated measurements, and it only requires steady state assumption for a relatively short period of time (60 to 90 minutes) to be valid. Therefore the bolus injection technique may be the preferred technique in critically ill patients.

In healthy individuals the endogenous glutamine production is in the order of 50 to 70 g per 24 h, corresponding to approximately 5 μmol/kg/minute [13]. It is only marginally influenced by intravenous nutrition or intravenous supply of exogenous glutamine [14]. So far only singular estimates from critically ill patients are reported, by the constant infusion technique [15] or by the decay curve technique [10,11]. These studies of critically ill subjects report glutamine R_a_ of levels comparable to those of healthy volunteers and of metabolically uncompromised patients scheduled for elective surgery [8,9,13].

In the present study, the recently presented bolus injection technique to estimate endogenous glutamine production, which enables us to make repetitive measurements, was applied to critically ill patients in the ICU. The primary aim was to estimate the possible impact of adding exogenous glutamine supplementation by an intravenous infusion of a glutamine containing dipeptide in a clinically relevant dose in the fed state on the endogenous glutamine production as estimated by the glutamine R_a_.

## Materials and methods

Patients (n = 11) on mechanical ventilation in the ICU were included in the study. Inclusion criteria were (i) mechanical ventilation, (ii) >18 years and (iii) expected to stay on mechanical ventilation in the ICU without any major alteration in treatment for the next 48 h. Exclusion criteria were (i) no informed consent and (ii) any withholding or withdrawing of treatment. As described in detail below patients were randomized to control before glutamine (n = 6, gender male/female (m/f) 4/2, body mass index (BMI) 25 to 39 kg/m^2^), or glutamine before control (n = 5, gender m/f 4/1, BMI 23 to 38 kg/m^2^). Additional characteristics of the patients at start of the study are given in Table 1. The protocol was approved by the Ethics Committee at Karolinska Institutet, Stockholm, Sweden, and informed consent was obtained from a close relative of the patient, as all patients were on sedation.

**Table 1:** Patient characteristics

**Patient number**	**Age (years)**	**Diagnosis**	**Days in ICU at study start**	**SOFA at study start**
1	58	Medical	13	14
2	56	Surgical	6	15
3	79	Medical	2	13
4	39	Medical	17	7
5	75	Medical	8	12
6	68	Neurological	2	6
7	69	Surgical	8	11
8	76	Surgical	2	11
9	84	Surgical	4	11
10	74	Medical	1	12
11	51	Medical	6	10

Patients 1 to 6 were randomized to measure in the fed state at baseline (control), before being fed and glutamine supplemented, patients 7 to 12 were randomized to measure in the fed state and glutamine supplemented before feeding at baseline (control). SOFA, sequential organ failure assessment.

**Figure 1:**
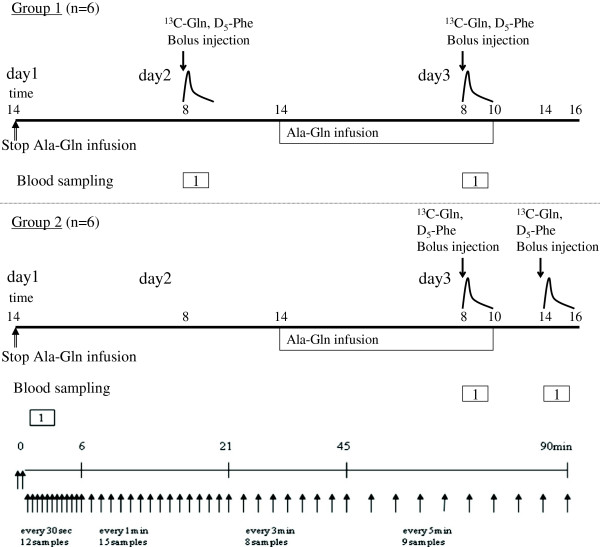
**The experimental protocol used.** Mechanically ventilated patients on full nutrition in the ICU (n = 11) were studied. They were randomized to measurement of basal endogenous glutamine R_a_ before (upper panel) or after (mid panel) exogenous intravenous glutamine dipeptide (Ala-Gln) supplementation. For the glutamine R_a_ measurements the ^13^C-glutamine bolus technique was employed. The post-bolus sampling schedule is depicted in the lower panel.

The protocol, illustrated in Figure 1, included two measurement periods in each patient. The inclusion criteria were chosen to recruit patients in a stable condition. Reference measurements were made before or after provision of exogenous glutamine in a crossover design. During the study period the basal nutrition, not containing any glutamine supplementation, was kept unaltered. So the patients were randomized to have a baseline measurement before or after a 20-h glutamine dipeptide infusion. The washout period after the end of the dipeptide infusion was 4 h, which has been demonstrated to be associated with an >80% decrease in the elevation of plasma glutamine concentration attributable to an ongoing intravenous glutamine supplementation [16].

Paired measurements were obtained from each patient during full nutritional support, but with and without intravenous exogenous glutamine supplementation. Nutritional support consisted of parenteral nutrition only (Kabiven®, Fresenius-Kabi, Bad-Homburg, Germany) or a combination of enteral (Fresubin®, Fresenius-Kabi) and parenteral nutrition. The nutritional target was 20 kcal/kg/24 h or energy expenditure as measured by indirect calorimetry. Extra glutamine was given by a constant intravenous infusion of 0.28 g glutamine/kg body weight during 20 h, given as an infusion of L-alanyl-L-glutamine, 200 mg/mL (Dipeptiven®, Fresenius-Kabi).

Each measurement period included a bolus injection of 1-^13^C-glutamine (3 mg/kg bodyweight; 99 atoms percent excess (APE) and ring-^2^H_5_-phenylalanine (0.3 mg/kg body weight; 99 APE), followed by frequent plasma sampling during 90 minutes (Figure 1) [12]. The doses were chosen to give sufficient increments and decay curves of the isotopic labels without any major influence on the plasma concentrations of glutamine and phenylalanine, respectively. The decay curves (See Additional file 1: Figures ES1 and ES2) allow for calculations of the R_a_ for glutamine and for phenylalanine by dividing the bolus injection by the area under the curve (AUC) up to 90 minutes for the tracer analyses in the blood by a single pool model,

$$R_{a}=\mathrm{Dose}/\mathrm{AUC}$$

In which Dose is the amount of the tracer injected and AUC is the area under the curve of the APE versus time. The exogenous given glutamine and phenylalanine from the dipeptide and nutrition were subtracted from the R_a_ to give estimates of the endogenous glutamine production, and of the endogenous phenylalanine production. As there is no endogenous *de novo* synthesis of phenylalanine, the latter gives an estimate of whole body protein degradation, which then enables a calculation of the fractions of endogenously produced glutamine that originates from protein breakdown and from *de novo* synthesis. For this calculation we assumed whole body protein contents of glutamine and phenylalanine of 7.0 and 4.2%, respectively [17].

Blood samples for analyses were drawn from an arterial line according to the protocol in Figure 1. To reduce the amount of blood needed for the two measurements the waste taken from the catheter before obtaining the real sample was given back to the patient in another intravenous catheter. Samples in ethylenediaminetetraacetic acid (EDTA) tubes were immediately put on ice, and centrifuged in a cool centrifuge within 30 minutes to obtain plasma, which was stored at -80°C pending analysis.

Plasma samples were analyzed for glutamine and phenylalanine enrichments and concentrations using gas chromatography-mass spectrometry (GC-MS) measurements as described before [17]. In short, plasma was deproteinized using methanol. Ammonium formate was added at this step to prevent conversion of glutamine to glutamate. Subsequently the supernatant was dried by rotary evaporation and the amino acids derivatized with N-methyl-N-tert-butyldimethylsilyltrifluoroacetamide (MTBSTFA) before analysis by GC-MS (Agilent 5379 N, Agilent, Solna, Sweden). To measure the concentration of glutamine an internal standard in the form of ^13^C_5_-glutamine was added and compared with a standard curve.

All analyses were performed using GraphPad Prism software (version 5, GraphPad Software, Inc. La Jolla, CA, USA). Comparisons between the basal and supplemented states were done by the Student *t*-test for paired samples. A probability value of *P* <0.05 was considered statistically significant.

## Results

**Figure 2:**
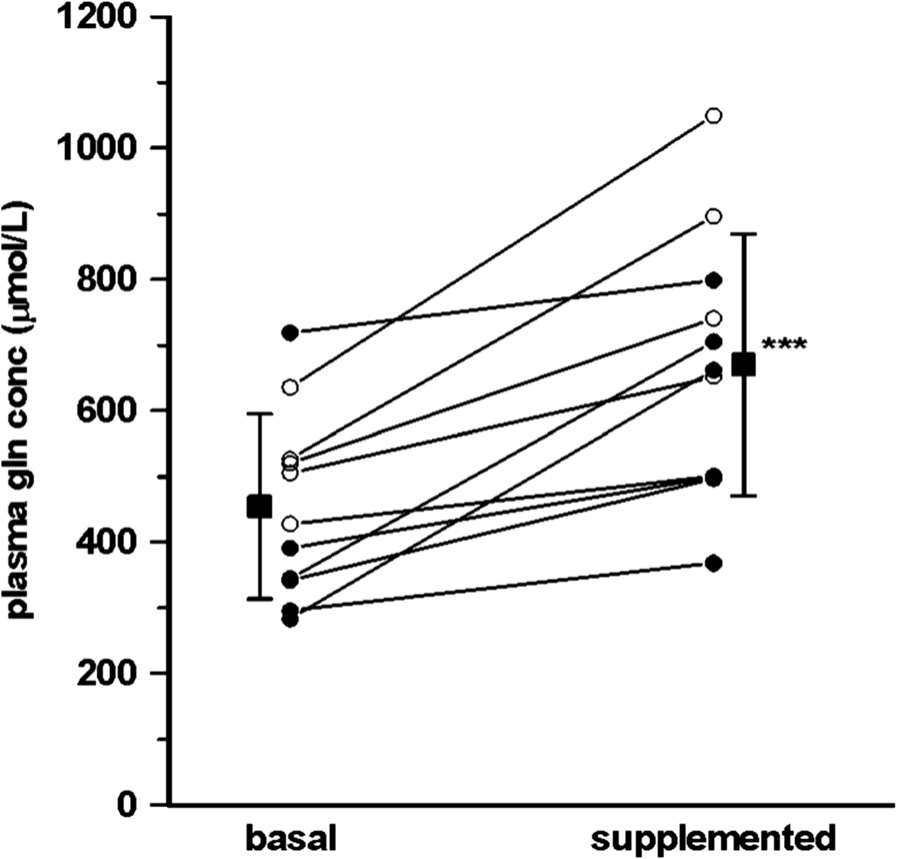
**Plasma glutamine concentration in mechanically ventilated patients on full nutrition in the ICU (n = 11).** The basal sample was taken immediately before the start of exogenous intravenous glutamine supplementation with L-alanyl-L-glutamine (filled symbols, n = 6), or after a washout period of 4 h following the termination of the intravenous glutamine supplementation with L-alanyl-L-glutamine (open symbols, n = 5). Mean values with SD are indicated by filled squared symbols. ***Iincrease in plasma glutamine concentration statistically significant, *P* = 0.0004.

Due to failure to detect any glutamine tracer in one of the measurements in one patient, results are only reported for 11/12 patients. Plasma glutamine concentration at the baseline fed state was 454 ± 141 μmol/L and at the end of the 20-h intravenous infusion of an exogenous glutamine containing dipeptide it had increased by 70% to 670 ± 199 μmol/L (*P* = 0.0004). All subjects studied showed an increase in the plasma glutamine concentration during the dipeptide infusion as compared to the baseline fed state (Figure 2).

**Figure 3:**
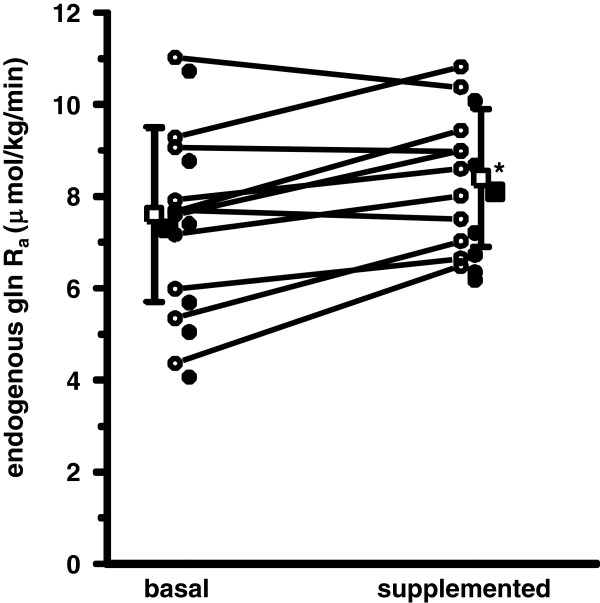
**Endogenous glutamine production in mechanically ventilated patients on full nutrition in the ICU (n = 11) as assessed by measurements of glutamine R**_**a **_**using the bolus injection technique employing **^**13**^**C-glutamine.** The basal measurement was performed immediately before the start of exogenous intravenous glutamine supplementation with L-alanyl-L-glutamine (filled symbols, n = 6), or after a washout period of 4 h following the termination of the intravenous glutamine supplementation with L-alanyl-L-glutamine (open symbols, n = 5). Mean values with SD are indicated by filled squared symbols. **Increase in glutamine R_a_ statistically significant, *P* = 0.009.

The experimental setup was to study the baseline fed state before treatment in half the study group and treatment before the baseline fed state in the remaining patients (Figure 1). No statistical differences in terms of plasma glutamine concentration (Figure 2) or glutamine R_a_ (Figure 3) attributable to the order of the measurements was detectable. Therefore the two study protocols are not separated in the results.

The glutamine R_a_ calculated from the decay curves of the isotopic labels (see Additional file 1: Figures ES1 and ES2), showed a 14% higher value (*P* = 0.009) during the last 1.5 h of the glutamine containing dipeptide infusion as compared to the baseline fed state (Figure 3). This corresponded to a numerically higher value of glutamine R_a_ in 8/11 subjects investigated during the dipeptide infusion. When the plasma concentrations of glutamine were correlated to the glutamine R_a_, no relationship was seen (*r*^2^ = 0.04, *P* = 0.43).

The phenylalanine R_a_ did not show any difference related to the glutamine containing dipeptide infusion (see Additional file 2: Figure ES3). With the assumptions made in the calculations, the whole body protein degradation then contributed approximately 25% of the total glutamine R_a_ in both the baseline fed state and during the glutamine containing dipeptide infusion. That left the whole difference in glutamine R_a_ between the baseline fed state and the fed state with an exogenous glutamine containing dipeptide infusion to the *de novo* synthesis (Table 2). The influence of the exogenously supplied glutamine in the enteral nutrition used was neglectable. Even with a 100% enteral nutrition of 1 kcal/kg/h the theoretical contribution from that source would be <0.1 μmol/kg/minute or <1.5% of the calculated glutamine R_a_. Therefore no corrections of the values have been made to compensate for the glutamine contained in the enteral nutrition given to some patients.

**Table 2:** **Endogenous rate of appearance (R**_**a**_**) for phenylalanine (Phe) and glutamine (Gln) and calculated origin of produced glutamine in critically ill patients (n = 11) fully fed, with and without intravenous glutamine supplementation**

	**Baseline fed**	**Fed and Gln supplemented**	***P*-value**
Endogenous R_a_ Phe (μmol/kg/minute)	1.22 ± 0.31	1.36 ± 0.52	0.326
Endogenous R_a_ Gln (μmol/kg/minute)	7.11 ± 1.90	8.08 ± 1.53	0.009
Gln from protein breakdown (μmol/kg/minute)	1.08 ± 0.27	1.20 ± 0.43	0.336
Gln from de novo synthesis (μmol/kg/minute)	6.02 ± 1.82	6.89 ± 1.24	0.025

Results are presented as mean **±** SD.

## Discussion

For the first time, repetitive measurements of glutamine R_a_ were performed in critically ill patients giving estimates of endogenous glutamine production. The hypothesis that exogenous glutamine supplementation with increased plasma glutamine concentration would attenuate endogenous glutamine R_a_ was rejected. On the contrary during an intravenous infusion of L-alanyl-L-glutamine in a clinically relevant dose a 14% increase in endogenous glutamine R_a_ was seen. It was demonstrated that this increase was confined to the *de novo* produced glutamine and not to the glutamine derived from protein degradation. Finally no relation was seen between plasma concentration and endogenous glutamine R_a_.

Reliable information on the endogenous production of glutamine and its relation to plasma glutamine concentration is crucial to explore the pivotal role of glutamine in critical illness. As an independent predictor of outcome at ICU admission, a low plasma glutamine concentration is an ominous sign [1,2]. The normalization of plasma glutamine is demonstrated by an intravenous supplementation of 0.2 to 0.3 g/kg/24 h leading to improved outcomes [18-20]. Nevertheless plasma concentration was not a good estimate of the endogenous glutamine production as demonstrated in this study. However, the subjects studied were much too few to enable any definite conclusions on this point.

The advantage of a technique that can be utilized for repetitive measurements is obvious. Not surprisingly critically ill patients most often exhibit a larger scatter in various parameters as compared to healthy individuals. Therefore a study design that can use the same subject for both control and treatment is preferable. The alternative necessitates a substantially larger number of patients if two different groups should be compared for a parameter with a large variability. Alternatively inclusion criteria may be narrowed, with a corresponding lower degree of generalizability as a consequence.

R_a_ as an estimate of endogenous production is not without problems. Initially the concept was introduced for glucose, which is produced by some tissues and utilized by other tissues. The transport is via circulating blood, making up a sampling pool that contains almost the entire endogenous production. For an amino acid like glutamine the total production is not as easily defined, and a part of the endogenous glutamine production may not be represented in the systemic circulation, escaping to be measured as a part of the R_a_. This is of course inherent to any isotopic technique relying upon plasma sampling. On the other hand, the production of glutamine that is made available for other tissues and transported via the circulation is an important aspect in critical illness and also what determines the plasma levels.

Quantitatively the results are comparable to those presented in other groups of critically ill patients; multiple organ failure [15], burns [21], and head trauma [10]. The endogenous production is rather on the high side as compared to healthy subjects, although the plasma concentrations are often low. This also fits very well with the observation that the efflux of glutamine from skeletal muscle is higher in critically ill patients as compared to healthy subjects [22].

The finding that glutamine R_a_ increased during L-alanyl-L-glutamine infusion was unexpected. One possibility is that the metabolization of the alanine part of the dipeptide ends up in *de novo* glutamine synthesis. This hypothetical explanation has been tested in healthy volunteers in the basal state by giving alanine only, which did not influence glutamine R_a_[12]. Still, that experiment did not take into account the simultaneous provision of full nutrition. The fact that intravenous glutamine supplementation results in a higher, but stable glutamine concentration after a few hours and the fact that the extra supplementation does not reduce the glutamine endogenous production means that the extra glutamine given is utilized by the critically ill patient. What tissues use the extra glutamine needs to be elucidated, but these are most likely the tissues with a high cell-turnover like the immune cells and the gut. The 14% increase in endogenous glutamine R_a_ during intravenous dipeptide infusion as compared to basal, both during full nutrition, must be interpreted with caution, as the condition during measurements were not isocaloric nor isonitrogenous. The result is sufficient to reject the hypothesis of a negative feedback from higher plasma glutamine concentration, but not sufficient to allow for more than speculation over why an actual increase was seen.

The strength of the present study is the repetitive measurements of glutamine R_a_ in critically ill patients, detecting a higher glutamine R_a_ during the glutamine containing dipeptide infusion. This finding would have been difficult to demonstrate if paired measurements had not been possible. Limitations of the present study are the fact that the measured glutamine R_a_ is an estimate of the endogenous glutamine production rate, and also the heavy dependence upon the correct baseline when calculating the AUC in the bolus injection technique. The latter may be overcome by employing a two-pool model, a suggestion that has to be validated.

The absence of a relationship between plasma glutamine concentration and the endogenous glutamine production rate in this pilot study calls for further exploration of the relationship between the two. The glutamine plasma concentrations in Figure 2 indicate that five patients had low values (<400 uml/L), but no patients had high values. Furthermore patients were studied on days 1 to 17 of the ICU stay (Table 1). Future studies should be more systematic concerning the spontaneous plasma glutamine level as well as the time course of glutamine kinetics during critical illness before any definite conclusion over the relationship between plasma level and endogenous glutamine production can be drawn.

## Conclusion

The bolus injection technique to measure glutamine R_a_ as an estimate of the endogenous production of glutamine in critically ill patients was demonstrated to be useful for repetitive measurements. An intravenous infusion of L-alanyl-L-glutamine in a clinically relevant dose as part of full nutrition did not attenuate endogenous glutamine production. No relationship between plasma glutamine concentration and the endogenous glutamine production was seen.

## Key messages

• The hypothesized reduction in endogenous glutamine production during intravenous glutamine supplementation in critically ill patients was not seen.

• There was no relationship between endogenous glutamine production and plasma glutamine concentration in critically ill patients.

• The bolus injection technique to measure glutamine rate of appearance was useful for repetitive measurements in critically ill patients.

## Abbreviations

Ala-Gln: Alanyl-glutamine; APE: atoms percent excess; AUC: area under the curve; BMI: body mass index; EDTA: ethylenediaminetetraacetic acid; GC-MS: gas chromatography-mass spectrometry; Gln: glutamine; MTBSTFA: N-Methyl-N-tert-butyldimethylsilyltrifluoroacetamide; Phe: phenylalanine; Ra: rate of appearance; SOFA: sequential organ failure assessment.

## Competing interests

None of the authors have any conflict of interest of declare for the present study.

## Authors’ contributions

Conception and design of study, interpretation of data, finalizing manuscript: MM, MS, OR, IT, ÅN, and JW. Acquisition of data: MM, MS, IT and JW. Analytical procedures and methods: MM and OR. Calculations and preparing manuscript: MM, OR, ÅN and JW. All authors read and approved the final manuscript.

## Authors’ information

Maiko Mori, MSc, PhD-student at the Division of Anaesthesia at CLINTEC, Karolinska Institutet. Olav Rooyackers, PhD, Professor of Anesthesia and Intensive Care Medicine at the Division of Anaesthesiology at CLINTEC, Karolinska Institutet. Marie Smedberg, MD PhD-student at the Division of Anaesthesia at CLINTEC, Karolinska Institutet, and resident at the Department of Anesthesia and Intensive Care Medicine, Karolinska University Hospital Huddinge. Inga Tjäder, MD PhD, Postdoctorial Fellow at the Division of Anaesthesia at CLINTEC, Karolinska Institutet, and senior consultant at the Department of Anesthesia and Intensive Care Medicine, Karolinska University Hospital Huddinge. Åke Norberg, MD PhD, Postdoctorial Fellow at the Division of Anaesthesia at CLINTEC, Karolinska Institutet, and senior consultant at the Department of Anesthesia and Intensive Care Medicine, Karolinska University Hospital Huddinge. Jan Wernerman, MD PhD, Professor of Anesthesiology and Intensive Care Medicine at the Division of Anaesthesia at CLINTEC, Karolinska Institutet, and senior consultant at the Department of Anesthesia and Intensive Care Medicine, Karolinska University Hospital Huddinge.

## Supplementary Material

Additional file 1 **Diagrams over the isotopic enrichments after a bolus injection of 1-**^**13**^**C-glutamine and ring-**^**2**^**H**_**5**_**-phenylalanine.** “Spagettigram”representations for all individual subjects using nominal and logarithmic scales. Click here for file

Additional file 2 **Diagram over whole body protein degradation.** A “spagettigram”representations for all individual subjects, employing a bolus injection of d5-phenylalanine to assess phenylalanine R_a_ as a measurement of whole body protein degradation. Click here for file
