# Supplementary material for: Endogenous glutamine production in critically ill patients: the effect of exogenous glutamine supplementation
Source: Crit Care. 2014 Apr 14;18(2):R72. doi: 10.1186/cc13829 (PMC4056090; doi:10.1186/cc13829)
Supplement: Additional file 1 — Diagrams over the isotopic enrichments after a bolus injection of 1-13C-glutamine and ring-2H5-phenylalanine. “Spagettigram”representations for all individual subjects using nominal and logarithmic scales. [file cc13829-S1.doc]

Additional file 1.

Endogenous glutamine production in critically ill patients, the effect of exogenous glutamine supplementation.

Maiko Mori, Olav Rooyackers, Marie Smedberg, Inga Tjäder, Åke Norberg, Jan Wernerman

Department of Anaesthesia and Intensive Care Medicine at Karolinska University Hospital Huddinge and Karolinska Institutet, Stockholm, Sweden

Figure ES1.


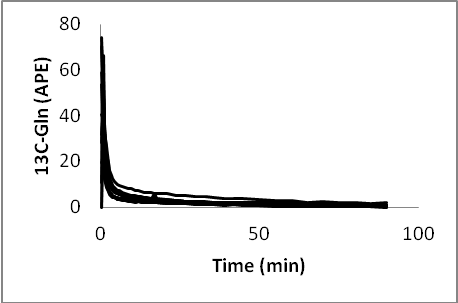

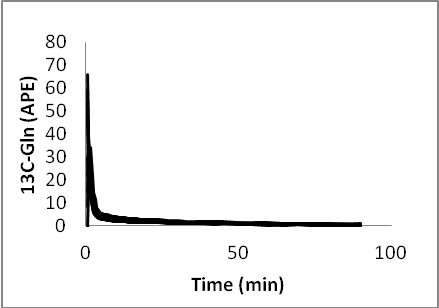

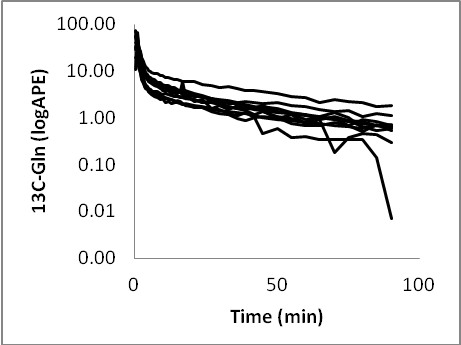

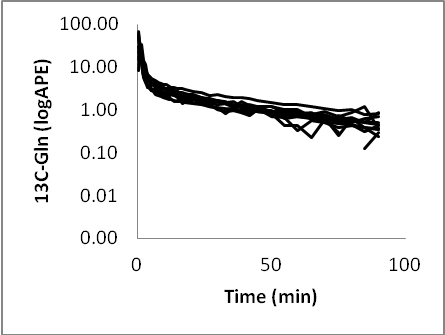


**Figure ES1.** The isotopic enrichments after a bolus injection of 1-^13^C-glutamine (3 mg/kg bodyweight; 99 APE) in fully fed critically ill patients (n=11), without exogenous glutamine supplementation (left hand panels), and during the last part of an intravenous infusion of 0.28 g glutamine / kg body weight during 20 hours, given as an infusion of L-alanyl-L-glutamine (right hand panels). Upper panels give the enrichments from 44 samples during 90 minutes on a linear scale, and to separate the individual curves that overlap on the linear scale, the same curves are given using logarithmic scales on the lower panels.

Figure ES2.


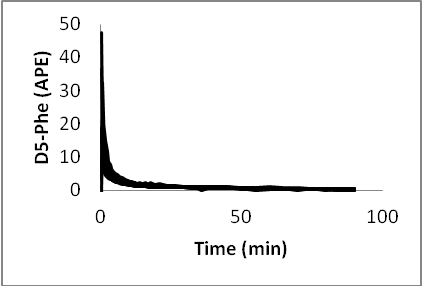

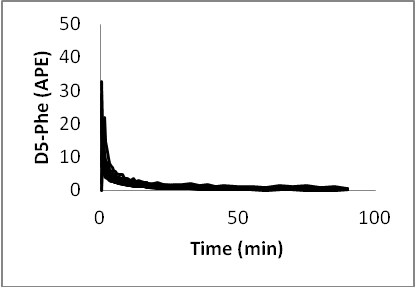

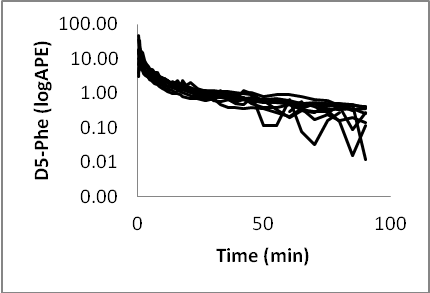

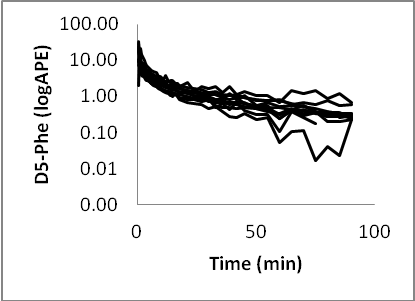


**Figure ES2.** The isotopic enrichments after a bolus injection of ring-^2^H_5_-phenylalanine (0.3 mg/kg body weight; 99 APE) in fully fed critically ill patients (n=11), without exogenous glutamine supplementation (left hand panels), and during the last part of an intravenous infusion of 0.28 g glutamine / kg body weight during 20 hours, given as an infusion of L-alanyl-L-glutamine (right hand panels). Upper panels give the enrichments from 44 samples during 90 minutes on a linear scale, and to separate the individual curves that overlap on the linear scale, the same curves are given using logarithmic scales on the lower panels.
