# Supplementary material for: Endogenous glutamine production in critically ill patients: the effect of exogenous glutamine supplementation
Source: Crit Care. 2014 Apr 14;18(2):R72. doi: 10.1186/cc13829 (PMC4056090; doi:10.1186/cc13829)
Supplement: Additional file 2 — Diagram over whole body protein degradation. A “spagettigram”representations for all individual subjects, employing a bolus injection of d5-phenylalanine to assess phenylalanine Ra as a measurement of whole body protein degradation. [file cc13829-S2.doc]

Additional file 2.

Endogenous glutamine production in critically ill patients, the effect of exogenous glutamine supplementation.

Maiko Mori, Olav Rooyackers, Marie Smedberg, Inga Tjäder, Åke Norberg, Jan Wernerman

Department of Anaesthesia and Intensive Care Medicine at Karolinska University Hospital Huddinge and Karolinska Institutet, Stockholm, Sweden

Figure ES3.

**Figure ES3.** Whole body protein degradation in mechanically ventilated patients on full nutrition in the ICU (n=11) as assessed by measurements of phenylalanine R_a_ using the bolus injection technique employing d5-phenylalanine. The basal measurement was performed immediately before the start of exogenous intravenous glutamine supplementation with L-alanyl-L-glutamine (filled symbols, n=6), or after a washout period of 4 hours following the termination of the intravenous glutamine supplementation with L-alanyl-L-glutamine (open symbols, n=5). Mean values with standard deviations are indicated by filled squared symbols. NS indicates that no change in phenylalanine R_a_ was detected, P=0.52.
